# Nucleic Acid Quantification by Multi-Frequency Impedance Cytometry and Machine Learning

**DOI:** 10.3390/bios13030316

**Published:** 2023-02-24

**Authors:** Mahtab Kokabi, Jianye Sui, Neeru Gandotra, Arastou Pournadali Khamseh, Curt Scharfe, Mehdi Javanmard

**Affiliations:** 1 Department of Electrical and Computer Engineering, Rutgers University, Piscataway, NJ 08854, USA; 2 Department of Genetics, Yale University School of Medicine, 333 Cedar Street, New Haven, CT 06520, USA; 3 Department of Mechanical and Aerospace Engineering, Rutgers University, Piscataway, NJ 08854, USA

**Keywords:** nucleic acid concentration, biosensor, machine learning, regression model, impedance cytometry, microfluidic chip

## Abstract

Determining nucleic acid concentrations in a sample is an important step prior to proceeding with downstream analysis in molecular diagnostics. Given the need for testing DNA amounts and its purity in many samples, including in samples with very small input DNA, there is utility of novel machine learning approaches for accurate and high-throughput DNA quantification. Here, we demonstrated the ability of a neural network to predict DNA amounts coupled to paramagnetic beads. To this end, a custom-made microfluidic chip is applied to detect DNA molecules bound to beads by measuring the impedance peak response (IPR) at multiple frequencies. We leveraged electrical measurements including the frequency and imaginary and real parts of the peak intensity within a microfluidic channel as the input of deep learning models to predict DNA concentration. Specifically, 10 different deep learning architectures are examined. The results of the proposed regression model indicate that an R_Squared of 97% with a slope of 0.68 is achievable. Consequently, machine learning models can be a suitable, fast, and accurate method to measure nucleic acid concentration in a sample. The results presented in this study demonstrate the ability of the proposed neural network to use the information embedded in raw impedance data to predict the amount of DNA concentration.

## 1. Introduction

DNA, the carrier of genetic information, is highly important in the biology and molecular electronics fields [1]. In addition to its biological role, DNA is a topic of significant interest with applications in nanotechnology, self-assembly, and structural flexibility, making it a subject of great interest [2,3,4]. Moreover, the DNA molecule is a source of rich electrical properties and has the potential to be used as a conducting material in electronic circuits [1]. Due to its electrical properties, we can utilize a multi-frequency lock-in amplifier (Zurich Instruments HF2A, Zurich, Switzerland) to measure the impedance response of beads coupled with different DNA amounts [1]. In this instrument, when a paramagnetic bead or particle passes through the sensing region, it interferes with the AC electric field between two electrodes, and consequently, a momentary increase in impedance can be observed [5]. Nowadays, an impedance-based cytometer can be implemented for the detection of bacteria, DNA amount per bead, cancer cells, and many other biological cells [5,6,7,8,9]. Many studies have shown the importance and application of microfluidic biosensors as a fast, reliable, and rapid platform for early-stage disease detection, as well as many other applications. For example, Mok et al. studied the development of a microfluidic platform to detect proteins [10]. Mahmoodi et al. developed a biosensor platform to detect cortisol in in small volumes of human serum [11]. The goal of this study was to create a cost-effective point-of-care and self-testing platform. Furniturewalla et al. developed a platform to count the number of blood cells from a pin-prick blood sample pipetted into a standard microfluidic PDMS chip [12]. Xie et al. developed a biomolecular sensing method that utilizes an array of nanoscale wells functionalized with antibodies. The method monitors changes in ionic resistance as the target protein binds inside the wells [13]. On the other hand, the development of microfluidic chips and experimental design often involves extensive investment and time effort, and it is prone to user bias. In this paper, we propose a machine learning (ML)-based model to address this difficulty.

Artificial intelligence (AI) has grown rapidly over the past decade and can be widely used in many aspects of biological information, ranging from drug discovery prediction to cancer prognosis [14,15,16,17,18]. Artificial intelligence employs a variety of statistical methods to detect and extract key features from complex datasets. In addition, AI provides a robust framework for creating feature representations from high-dimensional inputs and generalizing knowledge to new scenarios [19]. In recent years, the integration of machine learning methods with microfluidics has become a popular area of research. The combination of microfluidics, which generates large amounts of data, with machine learning for the analysis of these complex data sets represents a promising development in biotechnology [19,20,21,22,23,24]. To date, many studies have shown the application of machine learning to impedance cytometry. For example, Caselli et al. demonstrated the ability of neural networks to decipher impedance cytometer signals. They utilized an experimental dataset to predict single cell features, which were then used as inputs for classifier models [25]. Patel et al. applied a designed biosensor for detecting hemoglobin biomolecules with high sensitivity using polynomial regression models [26]. Schütt et al. applied a k-means algorithm for subpopulation clustering of peripheral blood mononuclear cells, based on peak voltage and phase [27]. As another example, Honrado et al. developed an ML-based method of classification of impedance data to distinguish and quantify cellular subpopulations at the early apoptotic versus late apoptotic and necrotic states [28]. Ahuja et al. used a support vector machine (SVM) classifier to discriminate between live and dead breast cancer cells by using the peak impedance magnitude and phase [29]. Feng et al. used fully connected networks to estimate three biophysical parameters based on the peak impedance amplitude at four frequencies, allowing them to classify five cell types [30]. Meanwhile, Sui et al. used a combination of multi-frequency impedance cytometry and supervised machine learning to classify particle barcodes [31].

Given the clinical significance of DNA, here we examine if a machine learning approach could facilitate and expedite the process of identifying the DNA amount per bead. In this analysis, six different concentrations of DNA, with a fixed length of 300 bp (base pairs), are coupled with 2.8 μm paramagnetic beads and passed through a custom-made microfluidic channel. Then, electrical measurements within the microfluidic chip are obtained to construct a machine learning model. The machine learning algorithm learns the relationship between the electrical measurements as an input and the DNA concentration per bead as an output. As a result, the machine learning approach could learn from historical data obtained from experiments to predict new output values [32]. With this technique, a trained model can be generalized to predict the DNA amount per bead for beads with an unknown DNA concentration. The objective of this study is to leverage the electrical measurements obtained from the Zurich Instruments tool, such as the frequency, peak intensity, and phase change of the peak intensity, to predict the DNA concentration. In this work, we proposed a novel regression approach to predict the amount of DNA by using electrical measurement features. To quantify the performance of the specified model, three types of machine learning approaches were constructed: classification, regression, and a hybrid model. In our analysis, we benchmarked 10 different deep learning architectures from simple to complex on four figures of merit (FOMs), namely, accuracy and error for the classification method, R_Squared, and the mean square error (MSE) of the regression model. Furthermore, we combined the best architectures from classification and regression to propose a novel hybrid regression model with an R_Squared value of 97%. The trained hybrid regression model may provide a general platform to predict the DNA amount per bead from electrical measurements obtained from the Zurich Instruments tool.

## 2. Materials and Methods

### 2.1. Experimental Setup

**Figure 1:**
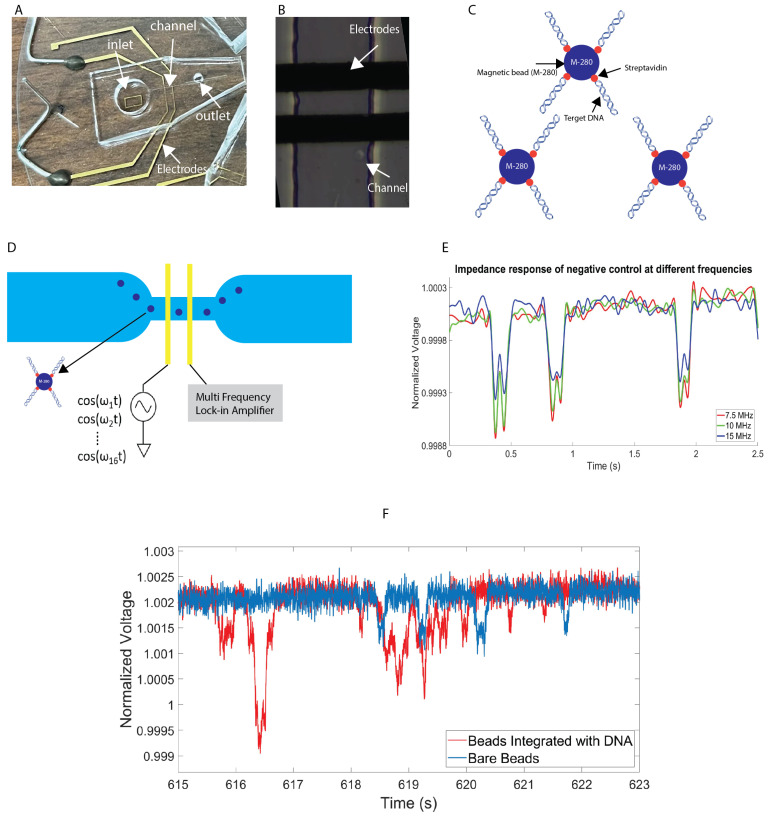
Overview of the process. (**A**) Image of device. (**B**) Microscopic image of channel and electrodes. (**C**) The sample preparation after binding of biotinylated DNA to paramagnetic beads. (**D**) The schematic diagram of detection. (**E**) Representative data of bare paramagnetic beads. (**F**) Representative data of bare paramagnetic beads and beads integrated with most-concentrated DNA.

As was described, nucleic acid quantification plays a major role in research and clinical study, ranging from the diagnosis of infectious diseases to food safety assurance and so on. Nucleic acids also have important biomarkers for biological studies and diagnosis [33]. In this experiment, a novel technique to identify DNA fragments is introduced. This technique identifies DNA fragments based on their frequency-dependent dielectric properties. In this experiment, DNA fragments which are coupled on micron-sized particles pass through a microfluidic channel made of polydimethylsiloxane (PDMS). The microfluidic PDMS channel is the first layer of the device. The second layer is a pair of electron beam-deposited reusable coplanar gold electrodes on a fused silica substrate. The microfluidic channel is 30 μm wide and 15 μm high, with a micron-sized electrode. The electrodes are 20 μm in width, and the gap between the two electrodes is 30 μm. We should point out that we experimentally verified that the sensitivity of the microfluidic channel increases as the width of the channel decreases and approaches the size of the bead. However, this increases the risk of clogging in the channel as it becomes too small. We designed the microchannel with the aforementioned configuration, which is large enough to minimize clogging and small enough to obtain sufficient sensitivity during measurements. Figure 1A represents the image of device which is made from PDMS and Figure 1B illustrates the microscopic image of the channel [5].

We compared our method to two commercially available technologies: gel electrophoresis and real-time PCR (also known as quantitative PCR or qPCR). Both of these are commonly used for DNA detection and sizing. The standard detection limit of gel electrophoresis using DNA bound to ethidium bromide is between 0.5 and 5.0 ng/band. However, with optimized gel electrophoresis technology, the Agilent Bioanalyzer can detect PCR products at concentrations as low as 0.1 ng/band and complete the analysis within 30 min. Real-time PCR has a detection limit of several copies of a DNA molecule per microliter or several fg/μL. However, it is relatively slow, with a sample processing time of over an hour, and has limitations in terms of DNA fragment size (e.g., amplicon size should be <200 bp). Furthermore, real-time PCR is costly and complex due to the need for simultaneous thermal cycling and fluorescence detection. It has limited multiplexing capabilities, making it difficult to miniaturize for portable applications. In contrast, our impedance sensor in combination with microfluidic technology has the potential for multiplexing and portability.

In this experiment, six different quantities and concentrations of DNA with a fixed length of 300 bp are integrated with a 2.8 μm paramagnetic bead and pass through a custom-made microfluidic chip. Three different types of magnetic beads (M270, M280, and C1) are tested. Based on the properties and the nature of our sensor, we chose to proceed with the M280 type (2.8 μm paramagnetic bead).

In this study, purified biotinylated DNA of a known quantity was serially diluted to obtain the desired concentrations. This DNA was then mixed with the beads to create DNA bound to the beads. The number of DNA molecules per bead is only an estimated average based on measurements of approximately 500 beads. This estimation was made after testing approximately 2000 beads per sample. The DNA-binding efficiency is determined by the very high binding affinity of the streptavidin–biotin interaction (K_d_ =$10^{-15}$). The beads contain streptavidin, and the DNA is biotin-labeled. These beads have a binding capacity of 10 ug ds-DNA per mg of beads. This knowledge was used when combining various DNA amounts with the beads. In this study, the lower limit of detection identified is 0.0039 fmol, and the maximum DNA concentration is 0.19 fmol [5]. For testing the sensitivity of the sensor, we diluted a 1-microliter aliquot of the DNA-coated beads in 60 μL of phosphate-buffered saline (PBS) for detecting small amounts of DNA. PBS, which has a relatively high salt concentration and high conductivity, has been shown to enhance the sensitivity of impedance measurements. Our sensor is capable of quantifying DNA fragments at high accuracy and precision at the femtomolar level and over a 100-fold dynamic range. Figure 1C represents the streptavidin–biotin linkage between DNA and beads. In this method, the target DNA was generated by using biotinylated DNA oligonucleotides and PCR (polymerase chain reaction) [5]. The procedures are as follows:

1. Biotinylated oligos was synthesized by IDT (Coralville, IA, USA), which is used to amplify different fragment sizes of DNA; in this case the fragment size is 300 bp.
2. The PCR product was purified by using a Qiaquick PCR purification kit to remove any unincorporated biotinylated oligos.
3. The PCR was eluted in water and quantified for immobilization to the streptavidin coated on 2.8 μm (M280) beads.
4. The purified biotinylated DNA was immobilized with beads in room temperature for 15 min using gentle rotation at 2000 rpm.
5. The biotinylated DNA-coated beads were separated on a magnet and washed subsequently.
6. Finally, the washed biotinylated DNA-coated beads were resuspended in 10 μL of water. Different concentrations of DNA were bound to paramagnetic beads (Table 1) [5].

Multi-frequency impedance cytometry techniques have been performed to detect the impedance difference of beads integrated with different amounts of DNA. The impedance response was measured at 8 different frequencies simultaneously by using a multi-frequency lock-in amplifier (Zurich Instruments HF2A, Zurich, Switzerland). When an AC voltage is applied between electrodes, a flowing particle or cell perturbs the AC electric field, which results in a momentary increase in the impedance/decrease in the voltage.

In this experimental setup, the first electrode is excited with combination of 8 frequencies ranging from 100 kHz to 20 MHz, and the second electrode is connected to the transimpedance amplifier. Figure 1E shows representative multi-frequency time series data of bare magnetic beads in voltage. The voltage is normalized for a straightforward comparison. For testing of 300 bp DNA beads, 6 different concentrations of DNA were measured to study the effect of the different amounts of DNA on the frequency. To compare the impedance response from different DNA concentrations, the impedance of bare beads with no DNA was measured in the same experiment. Figure 1F shows representative time series data comparing bare magnetic beads to DNA at the highest concentration (500 kHz frequency). In this figure, as well, the voltage is normalized for better comparison.

Table 1 showed the different concentrations of DNA coupled with paramagnetic beads. To compare the impedance response of different concentrations of DNA integrated with paramagnetic beads, we performed the same experiment with bare beads. In this experiment, 2.8 μm paramagnetic beads with no DNA concentration passed through the microfluidic channel, and the impedance response of a bare bead is obtained.

The results showed that there is positive relationship between DNA amounts per bead and the impedance peak response (IPR). As DNA concentration per bead increases, the IPR increases as well. These findings showed the positive correlation of DNA amounts attached to beads with IPR. In addition, increased DNA amounts resulted in a higher surface potential of the beads, which was associated with a larger impedance difference compared to the control bare bead. The details of the nucleic acid sample preparation and the impedance chip preparation, along with the experimental procedures, are those described in the work by Sui et al. [5].

As we described, in this experiment 6 different DNA concentrations coupled to paramagnetic beads are examined. In addition, it is very difficult to bind very small inputs of DNA to beads. Given the need for testing small DNA amounts in many samples, there is a utility for novel machine learning approaches for accurate and high-throughput DNA quantification. Furthermore, by proposing a general regression model, we can predict unknown DNA concentrations with a fixed length of 300 bp coupled to a bead. The combination of microfluidics, which generates vast amounts of complex data, with machine learning methods represents an emerging opportunity in biotechnology. On the other hand, the development of microfluidic chips and experimental design is expensive and time-consuming, and the method is prone to bias by the user. In the next section, we propose a novel hybrid regression model to address this difficulty. All the electrical properties obtained from the Zurich Instruments tools (including frequency, imaginary and real part of peak intensity) are leveraged to identify correlations between these properties and the amount of DNA per bead. Machine learning tools are then used to develop a general model and platform for predicting nucleic acid concentration.

### 2.2. Dataset

**Figure 2:**
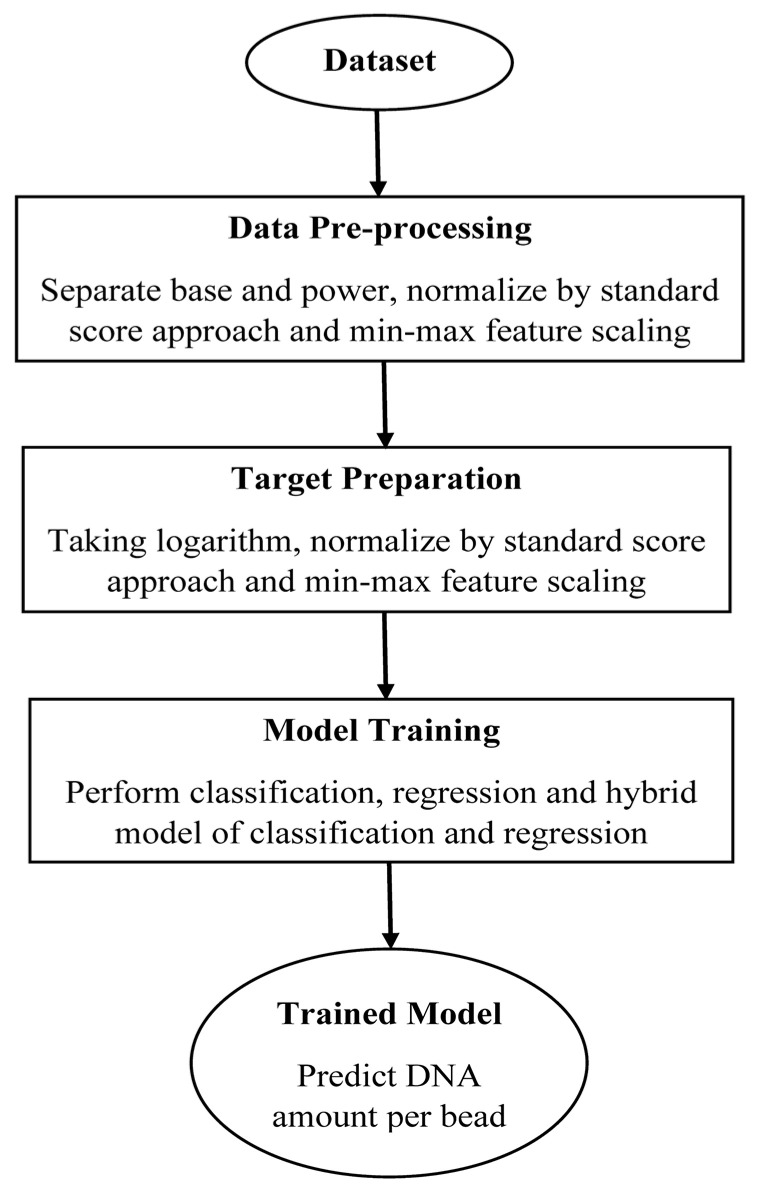
Overview of the proposed framework for prediction of DNA amount per bead.

This section explains the proposed approach to predicting the DNA amount per bead using experimental data and leveraging deep learning methods. Figure 2 shows an overview of the proposed method. In this study, the dataset was obtained from a custom-based microfluidic chip to detect DNA molecules bound to beads by measuring the impedance peak response (IPR) at multiple frequencies [5]. The proposed machine learning method will be trained on the electrical signals obtained from the biosensor with a specific configuration of the channel and electrode size. It is anticipated that increasing the size of the channel decreases the sensitivity of the biosensor. This means that the passage of beads or particles through the electrodes will result in weaker signals, i.e., smaller peaks in the impedance signals. This makes it more difficult to distinguish the passage of beads with very small amounts of DNA from the signal noise. Consequently, the accuracy of the machine learning method will be negatively affected. In the case of using another configuration, it would be more accurate to retrain the model based on the data obtained from the sensor with the new configuration.

The impedance response was measured simultaneously at 8 different frequencies ranging from 100 kHz to 20 MHz [5]. This dataset contains 105,104 data points collected on different days. For each piece of data, the frequency; the real, imaginary, and absolute values of the peak intensity; and the phase change of the peak intensity were measured to calculate the DNA amount per bead. All these features are used as input for the neural network model. In this work, our goal is to find a relationship between the aforementioned measurement features and the DNA amount per bead. To accomplish this, we explored three different machine learning approaches: classification, regression, and a hybrid model. The hybrid model is a combination of the best architecture of the classification and regression models.

The proposed model consists of three main steps, which are shown in Figure 2. The frequency; real, imaginary, and absolute values of the peak intensity; and the phase change of the peak intensity were recorded in measurements and will be used as input features. The output is the DNA amount per bead. In total, 7 outputs were examined containing 6 different concentrations of DNA from low to high coupled to paramagnetic beads and one control bead, which is a bare bead (i.e., a bead with no DNA concentration).

### 2.3. Data Preprocessing

The main goal of data preparation is to guarantee the quality of the data before applying them in any type of machine learning algorithm [34]. Before employing data in any learning algorithms, each input and output feature was normalized. Normalizing data generally prevents any variable from dominating the output values and boosts the accuracy of the model [35]. The most common normalization methods used in machine learning algorithms include min–max scaling, the standard score (z-score), and decimal scaling [36]. In this study, we applied two common normalization methods: the standard score and min–max scaling. First, we applied standard score normalization, and then we normalized the dataset between 0 and 1 (min–max scaling). In the standard score (z-score) normalization, the values for a feature *A* are normalized based on the mean (i.e., average) and standard deviation of *A* [34]. *A* value $v_{i}$ is normalized to $v{’}_{i}$ by computing:

(1) $${v’}_{i}={\frac{v_{i}-\bar{A}}{\sigma_{A}}}_{\;}$$

where $\bar{A}$ and $\sigma_{A}$ are the mean and standard deviation of attribute *A*, respectively [26]. Then, we applied min–max scaling normalization to our input features. In this technique, the attribute will be rescaled from its domain to a new range of values. In our case study, the input features are normalized in the (0, 1) range [36], where the following relation is used:

(2) $$f\left(v\right)=\frac{v-\min\left(v\right)}{\max\left(v\right)-\min\left(v\right)}{\;}_{\;}$$

Dataset normalization has a great effect on preparing the input data to be suitable for training and improving the accuracy of the output [35]. Many studies have employed more than one normalization method on input data before feeding data to any neural network’s algorithms to help comparing two or more datasets with different scales [37,38,39,40,41].

### 2.4. Target Preparation

Different quantities of DNA with a fixed length of 300 bp were tested in the experiment [5]. The output of our model is the DNA amount per bead for tested beads, including the bare bead and beads bonded with the least-concentrated DNA to those with the most-concentrated DNA, which are exponentially distributed. There is a total number of 7 outputs shown in Table 1. In situations where the data are distributed exponentially, taking a log function is one common way to normalize the data [42]. Therefore, we normalized the output features by using logarithmic transformation. In the next step, the standard score and min–max normalization are applied, which were described in Section 2.3.

### 2.5. Model Training

After feature extraction and preprocessing of data, 10 different deep learning architecture models are implemented to evaluate the performance of the approach. We employ classification, regression, and a hybrid model. The scikit-learn library is used to build the models in Google Colab using Python [43]. The data are shuffled randomly, and 30% is used for testing, while the rest is used for training. The model training was stopped after 5000 epochs (iterations) for feature selection, which is described in Section 3.1, and after 10,000 epochs for deep learning models both in classification and regression.

Before training the model, the most important task is to determine the combination of best features for DNA amount per bead prediction [40,44]. Moreover, the best number of features is chosen for regression and classification analysis. In each part of the analysis, 10 different models consisting of different numbers of hidden layers and neurons were implemented to examine the performance of different architectures. The best architecture giving the highest test and train accuracy and the lowest error was used as the best model for the classification part.

**Figure 3:**
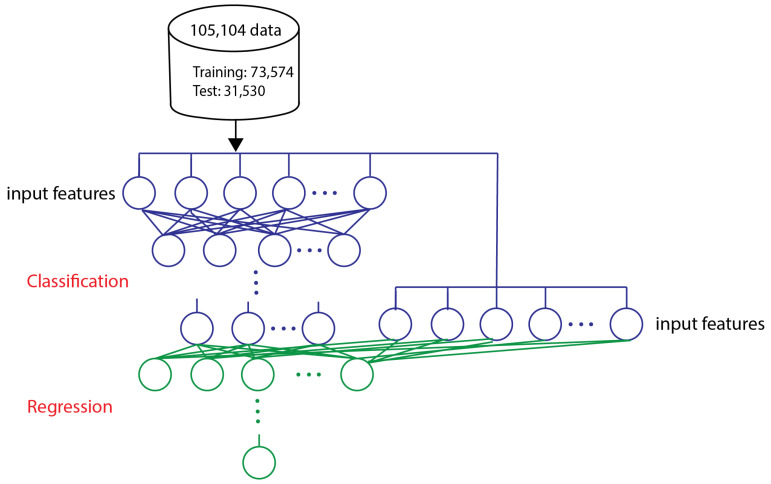
Hybrid model (combining the best architecture of the classification and regression models).

R_Squared and mean square error (MSE) are statistical parameters used to evaluate the performance of regression models [45]. The best deep learning architecture giving the highest R_Squared and lowest MSE was selected as the best candidate model. The hybrid model (Figure 3) uses the best architecture of the classification and regression models to train the model. The hybrid model is used to enhance the performance of the regression model. The prediction results of the classification model and original features are used as the input to the regression model. In other words, the 7 outputs from the classification method, combined with the 8 original input features, result in a total of 15 features that serve as the input to the candidate regression model. The output of the regression model is the DNA amount per bead.

## 3. Results

### 3.1. Feature Selection

**Figure 4:**
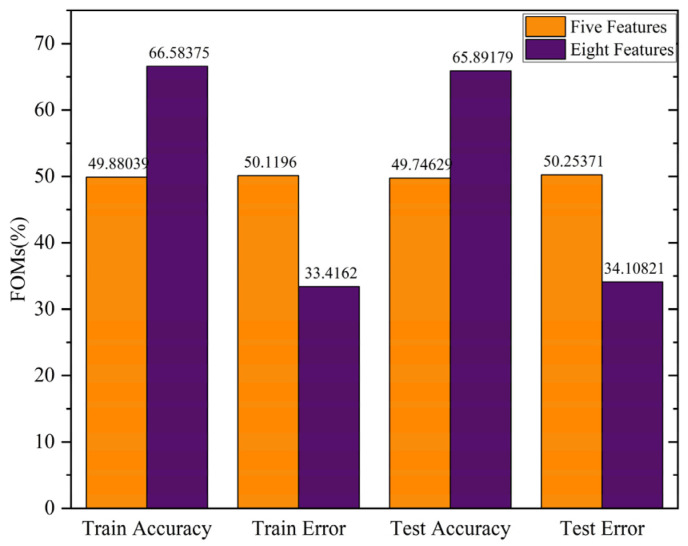
Effect of feature selection on FOMs (%).

We first studied the effect of the number of features on the performance of the classification and regression models, benchmarking their performance on four figures of merit (FOMs) in terms of accuracy and error for classification, and R_Squared and MSE for regression analysis. The model training stopped after 5000 epochs. The deep learning model consists of 5 hidden layers with 70, 60, 30, 20, and 10 neurons in each layer. For the input features, we evaluated two different datasets, including those with five and eight features. In the first dataset for DNA amount per bead prediction, five features consisting of frequency; the real part, imaginary part, and absolute value of the peak intensity; and the phase change of the peak intensity are used. For the second dataset, in addition to the frequency and phase change of the peak intensity, we divided each exponential input feature (real, imaginary, and absolute value of the peak intensity) into two parts: base and power. Figure 4 compares the performance of the classification model trained on the five-feature and eight-feature datasets. The results show that with the second dataset, which includes eight features, can lead to a more than 16% improvement in both training and testing accuracy. Furthermore, the train and test errors markedly decreased.

**Figure 5:**
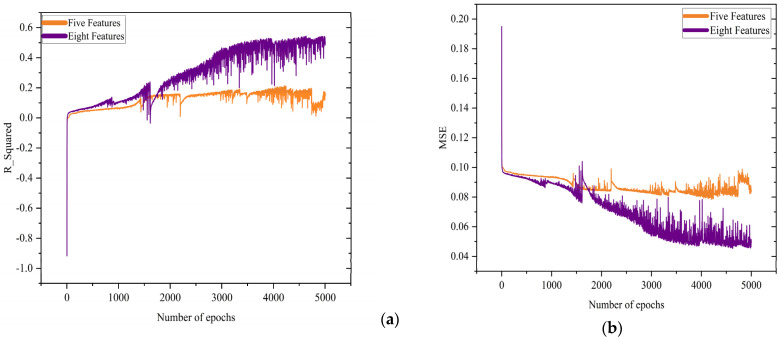
Effect of feature selection on (**a**) R_Squared with respect to number of epochs; (**b**) mean square error (MSE) with respect to number of epochs.

The effect of the number of features on the performance of the regression model was evaluated by the R_Squared and mean square error (MSE) values. The results are shown in Figure 5, which indicates that the dataset with eight features yields better results. Specifically, the regression model improved by around a 33% increase in R_Squared and around a 7% decrease in the MSE. Overall, both the classification and regression models performed better on representative FOMs; therefore, the dataset with eight features is chosen as the input for the following analysis.

### 3.2. Classification

To achieve robust network training, reduce the risk of overfitting, and increase the network generalization capabilities, we constructed 10 different deep learning architectures, from simple to complex. The implemented architectures are summarized in Table 2. Determining the optimal number of neurons and hidden layers is a very crucial step in deciding the optimal deep learning architecture [46]. Using too many neurons and hidden layers can result in overfitting by the model. On the other hand, having too few neurons and hidden layers may result in underfitting [46]. There are several methods and approaches to tuning the hyperparameters such as the number of neurons, activation function, number of layers, batch size, and epochs of deep learning algorithms. The possible approaches for finding the optimal parameters are hand or manual tuning, grid search, random search, Bayesian search, and AutoML. Grid search and random search are the most widely used strategies for hyperparameter optimization. In the grid search method, the domain of the hyperparameters is divided into a discrete grid, and the performance of every combination of values will be calculated. The point of the grid that maximizes the average value in cross-validation is the optimal combination of values for the hyperparameters [47]. While grid search evaluates the performance of every possible combination of hyperparameters to find the best model, random search only selects and tests a random combination of hyperparameters. Bergstra et al. [47] demonstrated that the performance of the random search is more efficient for hyperparameter optimization than trials on a grid. The Bayesian method, in contrast to random and grid search, builds a probability model to find the next set of hyperparameters which performs best on a probability function [48]. In other words, Bayesian optimization considers past evaluations when choosing the hyperparameter set to evaluate the next set of parameters [48]. All the aforementioned techniques are dedicated to special cases; as an example, grid search is only reliable for low-dimensional input spaces [47]. On the other hand, it was shown that random search results in better sampling efficiency in high-dimensional search spaces compared to grid search [49]. Bayesian optimization might potentially trap the model at a local optimum. In this analysis, manual tuning has been employed to determine the hyperparameters of the deep learning model to address these difficulties. In addition, manual tuning provides us the behavior of hyperparameters and reduces the runtime of the process. Therefore, we employed 10 architectures and analyzed the effect of the numbers of neurons and hidden layers on FOMs.

**Figure 6:**
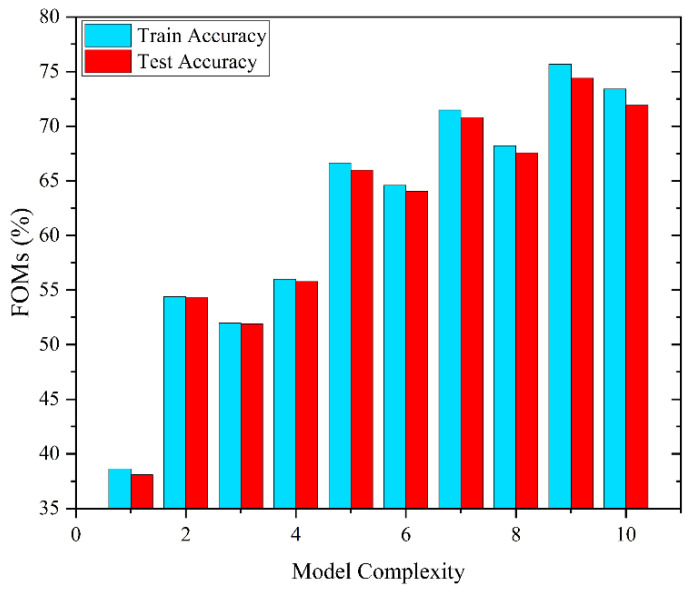
Effect of model complexity on train and test accuracy.

The test and train accuracy of each architecture are evaluated. The training procedure was stopped after 10,000 iterations for all models. In addition, the ReLU activation function was used, which is the most commonly used activation function in deep learning models. ReLU stands for rectified linear unit and is an activation function commonly used in neural networks. It is a simple function that outputs the input directly if it is positive and outputs zero if it is negative. Figure 6 represents the train and test accuracy of each architecture.

Among all architectures, model number 9 achieved the highest accuracy, which is around 75% on the training data and around 74% on the test data. It is also worth mentioning that the selected model performed well on train and test data. This means that the model generalizes well from observed data (train data) to predict unseen data (test data), and no overfitting occurs [50]. Therefore, we selected model number 9 as the representative model for classification. Table 3 shows the configuration matrix of the representative model. To evaluate the performance of the representative model, the following metrics are used: accuracy (ACC), true positive rate (TPR), true negative rate (TNR), false negative rate (FNR), and false positive rate (FPR). These measures are computed using the following equations:

(3) $$\mathrm{Accuracy}\;\left(\mathrm{ACC}\right)=\frac{\mathrm{TP}+\mathrm{TN}}{\mathrm{TN}+\mathrm{TP}+\mathrm{FN}+\mathrm{FP}}$$

(4) $$\mathrm{Sensitivity}\;\left(\mathrm{TRP}\right)=\frac{\mathrm{TP}}{\mathrm{TP}+\mathrm{FN}}$$

(5) $$\mathrm{Specificity}\;\left(\mathrm{TNR}\right)=\frac{\mathrm{TN}}{\mathrm{TN}+\mathrm{FP}}$$

(6) $$\mathrm{Fallout}\;\left(\mathrm{FPR}\right)=\frac{\mathrm{FP}}{\mathrm{TN}+\mathrm{FP}}$$

(7) $$\mathrm{False}\;\mathrm{Negative}\;\mathrm{Rate}\;\left(\mathrm{FNR}\right)=\frac{\mathrm{FN}}{\mathrm{TP}+\mathrm{FN}}$$

where TPs (FPs) refer to the number of correct (incorrect) predictions of outcomes in the considered output class, whereas TNs (FNs) refer to the number of correct (incorrect) predictions of outcomes in any other output classes [14]. The below table shows the accuracy (ACC), true positive rate (TPR), true negative rate (TNR), false positive rate (FPR), and false negative rate (FNR) for each individual output (class). For each individual class, we achieved above 88% accuracy.

### 3.3. Regression

**Figure 7:**
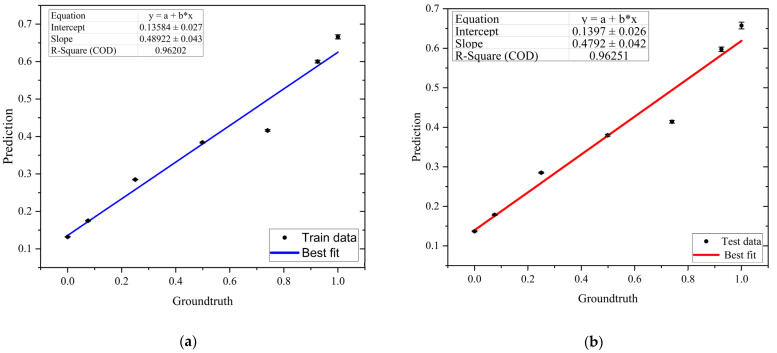
Results of representative regression model on (**a**) train and (**b**) test data.

Similar to classification, we constructed 10 different neural network architectures using the same models previously shown in Table 2. Table 4 displays the R_Squared and MSE of each model. According to the results given in Table 4, it can be concluded that among all architectures, model number 8 achieved the highest R_Squared for both the train and test data. Therefore, model number 8 is selected as the representative architecture for the regression model. Figure 7 shows the results obtained by the representative regression model on the test and train data. In this figure, the average DNA amount per bead prediction for each of our seven outputs is plotted versus its corresponding ground truth. The first point in Figure 7 represents the bare bead prediction, and the next six points represent the beads coupled with DNA concentrations from the lowest to the highest. This figure shows that there is a relationship between electrical measurements and DNA concentrations coupled to paramagnetic beads.

A linear fit was applied to these results, and an R_Squared of around 96% is achieved for both the train and test data, with a maximum standard error of 0.008. For an ideal model, the slope of the trend line should be equal to one, as the prediction should be equal to the ground truth. Here, the slope of trend line is around 0.47 indicating the error between the prediction and ground truth values. This motivated us to design a hybrid model to improve the performance of the regression model. In the next section, the architecture of the proposed hybrid model will be discussed.

### 3.4. Hybrid Model

The hybrid model shown in Figure 3 consists of the representative models of classification and regression combined together to increase the accuracy of the regression model. Model number 9 from the classification models (Table 2) is selected to be combined with the representative regression model (model number 8 from Table 2). In the resulting model, the output neurons of the classification model and original features are used as input features for the regression model. In this case, the eight aforementioned features were fed into the representative classification architecture, which resulted in seven categorical outputs. Then, these seven outputs with the eight original features served as the inputs of the candidate regression model. Finally, the regression model output is the DNA amount per bead.

**Figure 8:**
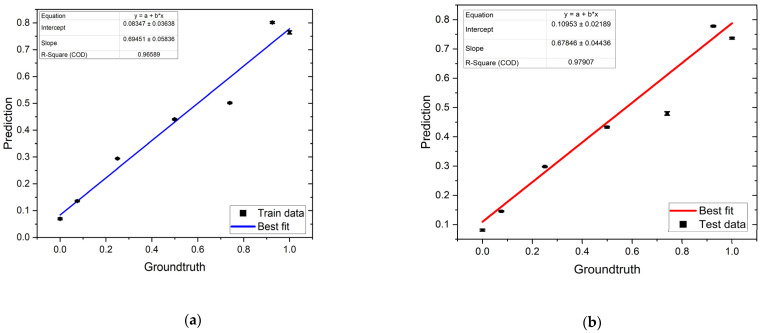
Representative hybrid model on (**a**) train and (**b**) test data.

The hybrid model is used to enhance the performance of regression. Figure 8 shows the results of the hybrid model on the train and test data, with an R_Squared of around 97%, a slope of around 0.68, and a maximum standard error of 0.005. Similar to Figure 7, the average DNA amount per bead prediction of each output class is plotted versus its corresponding ground truth. Comparing the hybrid model (Figure 8) and regression model (Figure 7), it can be seen that the slope of the model is improved by around 21%, by knowing the fact that the ideal slope is 1. In addition, the R_Squared value for the train and test data is improved.

To date, many studies have shown the effectiveness of using a hybrid model to enhance the performance of various systems. For instance, Liaqat et al. [44] proposed a hybrid model approach that combines seven classification algorithms with deep learning models to identify posture detection. In this study, the outputs of the ML classifiers and deep learning models were used as inputs for a convolutional neural network (CNN) architecture. The experimental results demonstrated that the proposed hybrid approach resulted in a better performance compared to traditional machine learning methods [44]. Chieregato et al. [51] also proposed a hybrid model that integrates machine-learning with deep learning methods and is designed to be used as a tool to support clinical decision-making. The proposed hybrid model is capable of predicting COVID-19 outcomes from CT images and clinical data. The reason for combining several state-of-the-art algorithms to build hybrid models is to enhance the accuracy of the model and increase its capability to tolerate significant data incompleteness [52]. However, complexity arises when one or more deep learning algorithms are combined, so careful consideration needs to be given to the selection of algorithms with different architectures to achieve better performance. Compared to conventional models, the hybrid model may take longer to train or tune.

By employing a hybrid regression model on data from impedance cytometry measurements of DNA, we have observed an 8% improvement in R_Squared compared to the linear regression model reported by Sui et al. [5]. The results presented in this work demonstrate the ability of the proposed neural network to use the information embedded in raw impedance data to predict the amount of DNA concentration coupled to beads. Artificial intelligence (AI) approaches provide a promising new direction to efficiently extract the information embedded in the electrical signals. From an application point of view, machine learning algorithms enable the development of intelligent microfluidic platforms. These platforms are operated by data-driven models and characterized by increased automation [19]. The results presented in this work demonstrate the ability of neural networks to efficiently predict the amount of immobilized DNA that is fixed in 300 bp. In developing our methods, three network types were considered: classification, regression, and a hybrid model. After selecting the best features, we constructed classification and regression models with optimized numbers of hidden layers. In the next step, a hybrid model was presented to improve the R_Squared of the model. The use of AI in analyzing impedance signals could present new challenges and opportunities for next-generation impedance cytometry systems.

## 4. Conclusions

In this study, we used a machine learning approach to predict the DNA amount per bead by leveraging electrical measurements from a Zurich Instruments tool. Multi-frequency impedance cytometry was performed to measure the electrical impedance responses at 8 different frequencies, ranging from 100 kHz to 20 MHz. In this experiment 6 different DNA concentrations were coupled to paramagnetic beads and passed through the microfluidic channel. To account for device-to-device variation, the response of bare streptavidin-coated paramagnetic beads was studied.

In the next step, we employed data from impedance cytometry measurements of DNA immobilized on paramagnetic beads to develop deep learning methods that can predict the amount of immobilized DNA that is fixed in 300 bp. The dataset used in this study consists of around 105,000 pieces of data with five electrical features. As a first step, we performed feature selection to identify the best combination of features. It was shown that when the base and power for the real, imaginary, and absolute values of the peak intensity were separated, better performance was achieved. Therefore, we continued our analysis using eight features.

In the next step, three different machine learning methods were presented, namely, classification, regression, and a hybrid model, to predict the DNA amount per bead. For classification and regression, underfitting and overfitting were studied by investigating 10 different deep learning architectures. For both classification and regression problems, the architecture with the highest performance was selected as the representative model. We were able to achieve around 75% accuracy for classification and an R_Squared of around 96% for regression. For the regression model, the average prediction values were plotted against ground truth, with a slope of 0.47 for the trend line.

To improve the performance of the regression model, a novel hybrid regression model was presented. In this approach, the best deep learning architectures for classification and regression were combined to predict the DNA amount per bead. The results showed that the proposed hybrid approach achieved a better performance as compared to the previous representative of regression models. In comparison to the regular regression model, the slope of the trend line improved by around 21%. The outcomes presented in this study demonstrate the ability of the proposed neural network to use the information embedded in raw impedance data to predict the DNA concentrations coupled to beads.

In future work, the focus will be on using automotive approaches to tune hyperparameters of deep learning methods, such as grid search, random search, and Bayesian search. The hybrid model has a longer training runtime than traditional machine learning algorithms, so further improvement and optimization are necessary to reduce the time cost. Additionally, testing different configurations of microfluid channels in terms of size and structure will be considered to assess the impact on the model’s performance and create a more generalized model.

## Figures and Tables

**Table 1:** Model outputs [5].

DNA Length	DNA Amount per Bead
Bare bead	0
300 bp	$1.54\times 10^{-4}$
300 bp	$7.69\times 10^{-5}$
300 bp	$1.54\times 10^{-5}$
300 bp	$1.54\times 10^{-6}$
300 bp	$1.54\times 10^{-7}$
300 bp	$1.54\times 10^{-8}$

**Table 2:** Deep learning models.

Model Number	Number of Hidden Layers	Number of Neurons in Each Layer
1	2	10,10
2	2	20,20
3	3	20,20,10
4	3	30,20,10
5	4	40,30,20,10
6	5	60,50,30,20,10
7	5	70,50,40,20,10
8	5	80,60,40,30,20
9	6	100,80,60,50,20,10
10	6	100,80,80,60,30,20

**Table 3:** Confusion matrix of representative model.

	1	2	3	4	5	6	7
ACC	0.97	0.91	0.89	0.89	0.88	0.97	0.97
TPR	0.43	0.87	0.80	0.87	0.51	0.8	0.78
TNR	0.99	0.92	0.92	0.89	0.93	0.99	0.99
FPR	0.004	0.07	0.07	0.10	0.06	0.07	0.09
FNR	0.56	0.12	0.19	0.12	0.48	0.99	0.21

**Table 4:** Effect of model complexity on R_Squared and MSE.

Model	MSE Train	MSE Test	$R^{2}$ Train (%)	$R^{2}$ Test (%)
1	0.3077	0.3077	67.61	67.98
2	0.2959	0.2954	57.34	56.83
3	0.2821	0.2873	72.64	71.26
4	0.2796	0.2811	75.39	74.76
5	0.2615	0.2786	90.69	90.6
6	0.2286	0.2481	93.34	93.13
7	0.2281	0.2373	91.89	92.16
8	0.2254	0.232	96.2	96.25
9	0.2117	0.2338	94.01	94.29
10	0.2087	0.2198	95.23	95.07

## Data Availability

The data presented in this study are available on request from the corresponding author. The data are not publicly available due to ethical constraints.
